# Diffusion Microscopist Simulator: A General Monte Carlo Simulation System for Diffusion Magnetic Resonance Imaging

**DOI:** 10.1371/journal.pone.0076626

**Published:** 2013-10-10

**Authors:** Chun-Hung Yeh, Benoît Schmitt, Denis Le Bihan, Jing-Rebecca Li-Schlittgen, Ching-Po Lin, Cyril Poupon

**Affiliations:** 1 Institute of Neuroscience, National Yang-Ming University, Taipei, Taiwan; 2 NeuroSpin, Commissariat à l’énergie atomique et aux énergies alternatives (CEA Saclay), Gif-sur-Yvette, France; 3 Institut de Federatif de Recherche 49, Gif-sur-Yvette, France; 4 Détermination de Formes et Identification (Equipe DEFI), Institut national de recherche en informatique et en automatique (INRIA Saclay), Palaiseau, France; University of Maryland, College Park, United States of America

## Abstract

This article describes the development and application of an integrated, generalized, and efficient Monte Carlo simulation system for diffusion magnetic resonance imaging (dMRI), named Diffusion Microscopist Simulator (DMS). DMS comprises a random walk Monte Carlo simulator and an MR image synthesizer. The former has the capacity to perform large-scale simulations of Brownian dynamics in the virtual environments of neural tissues at various levels of complexity, and the latter is flexible enough to synthesize dMRI datasets from a variety of simulated MRI pulse sequences. The aims of DMS are to give insights into the link between the fundamental diffusion process in biological tissues and the features observed in dMRI, as well as to provide appropriate ground-truth information for the development, optimization, and validation of dMRI acquisition schemes for different applications. The validity, efficiency, and potential applications of DMS are evaluated through four benchmark experiments, including the simulated dMRI of white matter fibers, the multiple scattering diffusion imaging, the biophysical modeling of polar cell membranes, and the high angular resolution diffusion imaging and fiber tractography of complex fiber configurations. We expect that this novel software tool would be substantially advantageous to clarify the interrelationship between dMRI and the microscopic characteristics of brain tissues, and to advance the biophysical modeling and the dMRI methodologies.

## Introduction

Diffusion magnetic resonance imaging (or dMRI) came into existence in the mid-1980s [1], [2], [3], and during the past 25 years, dMRI has been extraordinarily successful, particularly in MRI of the central nervous system. Its major clinical domain of application has been neurological disorders, especially for the management of patients with acute ischemic stroke. It is also rapidly becoming a standard for white matter (WM) disorders, as diffusion tensor (DT) imaging can reveal abnormalities in WM fiber structure and provide outstanding maps of brain connectivity [4], [5]. More recently, it has been shown that dMRI can also be used to deliver direct features of tissue microstructures [6], [7], [8], [9], as well as to detect changes in brain regions associated with neuronal activation [10]. The driving force of dMRI is to monitor natural microscopic displacements of water molecules that occur in brain tissues as part of the physical diffusion process. In other words, water molecules are used as a probe that can reveal microscopic details about tissue architecture, either in a normal or diseased state.

One has to keep in mind that the overall signal observed in dMRI images at a *millimetric* resolution results from the integration (on a statistical basis) of all the *microscopic* displacement distributions of the water molecules present in this voxel. The complex diffusion processes that occur in a biological tissue on a voxel scale are often described with a global and statistical parameter, the apparent diffusion coefficient (ADC) [2]. This parameterization of diffusion process by a global ADC is intended to represent those physical processes that occur at scales smaller than the scales resolved by the dMRI approach: The large scale is imposed by technical limitations (e.g. MRI hardware), while the actual “theatre” scales of the biophysical elementary processes are determined by physical phenomena at a molecular level. The averaging or smoothing effect resulting from this scaling presumes some homogeneity in the voxel and makes a direct physical interpretation out of the global parameter somewhat difficult, unless some assumptions can be made. The ADC in the brain is 2 to 10 times smaller than free water diffusion in an aqueous solution [11]. Such reduction has been explained by the effects of high viscosity, macromolecular crowding, and restriction in the intracellular space [12], and the effect of tortuosity in the extracellular space [13], . Restricted diffusion effects, for instance, may be evaluated by changing the diffusion time [15], [16]: Since the displacements of the molecules become confined when they reach the boundaries of closed spaces, the diffusion coefficient artificially goes down with longer diffusion times. Likewise, in brain tissues cell membranes likely hinder the water diffusion process (so-called “hindered” diffusion, as opposed to strictly “restricted” diffusion), even though the membranes are permeable to water via either passive or active transport, such as the specific aquaporin channels that have been found abundant in the brain [17]. Clearly water diffusion in biological tissues, especially the brain, is not free and cannot be modeled by a single Gaussian distribution [18]. Moreover, the ADC depends not only on the actual diffusion coefficients of water molecular populations presenting in an MRI voxel, but also on the experimental and technical parameters of MRI, such as the voxel size, the diffusion time or the degree of sensitization of MR images to diffusion (i.e. *b*-value) [2].

Although the idea to infer microstructures of brain tissues from dMRI signal alone is ill-posed, except in specific and simple situations, the relationship between ADC and certain tissue microscopic features is the object of intensive research. Some groups have tried to clarify how tissue characteristics affect the dMRI signal [19], [20], [21], [22], [23]. Theoretical models have been proposed, for instance, based on a combination of extra-axonal water undergoing hindered diffusion and intra-axonal water undergoing restricted diffusion [24]. Several groups have also underlined the important roles of dynamic parameters, such as membrane permeability and water exchange [25], [26], [27], as well as geometrical features, such as cell size distribution or axons/dendrite directional distribution [20], [26], [28], [29]. However, it is noticeable that all of those distinct models require strong assumptions to be made about the tissue structure or property, which may not always match known or unknown biological reality.

Another approach is to rely on Monte Carlo (MC) simulations, which have been shown to be a powerful and flexible tool to mimic diffusion processes for a wide class of systems, especially when analytical solutions cannot be obtained due to the complexity of the systems. Analytical approaches predicting the dMRI signal using the Bloch-Torrey equation, for example, must rely on simplified biological tissue model and MRI pulse sequence (e.g. rectangular gradient waveform) [30]. In realistic situation, however, the biological microstructures are too complicated to solve analytically; meanwhile, the difficulty in deriving solutions may be further increased following the complexity of the design of MRI pulse sequence and gradient profile. The advantage of the MC approach is its ability to track dynamic events over space and time for a system with many coupled degrees of freedom. It provides opportunities to investigate the Brownian motion in an arbitrary environment as well as any model of interaction between spins and membranes. Hence, synthetic dMRI data generated using the MC method can be applied to study biological properties (e.g. cell size, density, and membrane permeability) and basic diffusion mechanisms in different compartments (e.g. presence of attractors, local viscosity, and membrane interactions). On the one hand, it can be adapted to examine mechanistic hypotheses for various dynamic scenarios and tissue models, such as acute ischemia or neuronal activation and cell swelling, cancer and cell proliferation, ADC and axonal fiber anisotropy in complex fiber bundles or cortex. On the other hand, it has the potential to build a ground-truth database to support the development and application of dMRI studies (see below).

One of the most outstanding contributions and applications of dMRI is its ability to visualize anatomical connections between different brain areas, non-invasively and on an individual basis, which has emerged as a major breakthrough for neurosciences [31], [32], [33], [34]. There are two essential procedures to create a reliable map of brain connectivity using so-called fiber tractography: The first step is to accurately estimate fiber orientations using an adequate diffusion reconstruction method, and the second step is to implement a robust fiber-tracking algorithm. Obviously, it is necessary to have an appropriate model to serve as a “gold standard” for assessment and validation of these processes. Several evaluation models have been proposed and can be categorized into three main groups: (i) An animal model, such as using manganese ion as a tract tracer [35], [36], enables MRI experiments in real biological tissues, however, it lacks the plasticity to tune structural or geometric parameters (e.g. fiber curvature). Furthermore, the WM structures of animal models cannot capture all of the fiber configurations that exist in human brains, and thus it may not be sufficient to evaluate the inherent limitations of diffusion models and fiber-tracking algorithms. (ii) A physical phantom is able to provide experimental datasets acquired using practical dMRI setting and is more flexible than an animal model in terms of the geometric designs. The physical phantoms can be broadly classified into two types according to material: the hollow capillary [37], [38], [39], [40], [41] and the synthetic fiber [42], [43], [44], [45]. The advantage of the former is that it has a diameter closed to the scale of axonal fibers (∼10 µm) and is able to capture the nature of intra- and extra-axonal diffusion. However, it is not feasible to build complex configurations (e.g. bending fibers). On the contrary, the latter is highly flexible to construct curving structures similar to WM fibers, whereas it is limited to simulate the extra-axonal compartment. Note that both of the above materials lose the properties of biological tissues, such as membrane permeability and local viscosity. (iii) Numerical simulations have been widely chosen to generate synthetic dMRI datasets [46], [47], [48], [49], however, most of the numerical simulations usually rely on a number of assumptions on tissue models and pulse sequences. The Gaussian mixture model, for example, is commonly used to generate diffusion-weighted (DW) signal. Although the tensor model provides a good approximation, the Gaussian assumption is not sufficient to model diffusion anisotropy observed in living tissues, which is generally acknowledged to the results of restrictions and hindrances to the movements of water molecules [24]. Furthermore, the underpinning mechanism of water diffusion in neural tissues is actually more complicated if the cell membrane properties were considered [50]. By contrast, simulations based-on MC method has the potential to remove most of the assumptions inherently required by the numerical simulations described above. In addition, it is capable of simulating biological characteristics and configurations at different levels of complexity (e.g. mixture fiber radii or cell membrane permeability). Note that, importantly, the intrinsic challenge of MC approach is that it requires an adequate sample size (i.e. the number of random walkers and steps) in order to ensure the stability and reliability of the simulation results. Hence, in terms of software practicability, it is necessary to optimize MC simulation programs to achieve reasonable computation efficiency for a commonly available computing power.

### Computing Performance

The flexibility and plasticity of MC method strongly motivated us to develop a novel MC simulation system for dMRI, named Diffusion Microscopist Simulator (DMS), which has the ability to generate 3D tissue models of various shapes and properties, as well as to synthesize DW images using a variety of MRI methods and pulse sequence designs. Additionally, DMS is implemented with parallel processing structure that allows distribution of computations on a grid of computers for high performance computing. DMS aims at (i) bridging the gap between elementary diffusion processes occurring at a micrometer scale and the resulting diffusion signal measured at millimeter scale, providing better insights into the features observed in dMRI (e.g. variation of ADC and diffusion anisotropy with cell size distribution), and (ii) offering ground-truth information for optimization and validation of dMRI acquisition schemes for different applications (e.g. fiber-tracking algorithm, diffusion reconstruction model, and microscopic dMRI). In the following sections, we describe the design and the key modules of DMS, and then demonstrate the potential applications of DMS via four benchmark experiments.

## Methods

### General Overview of DMS

**Figure 1:**
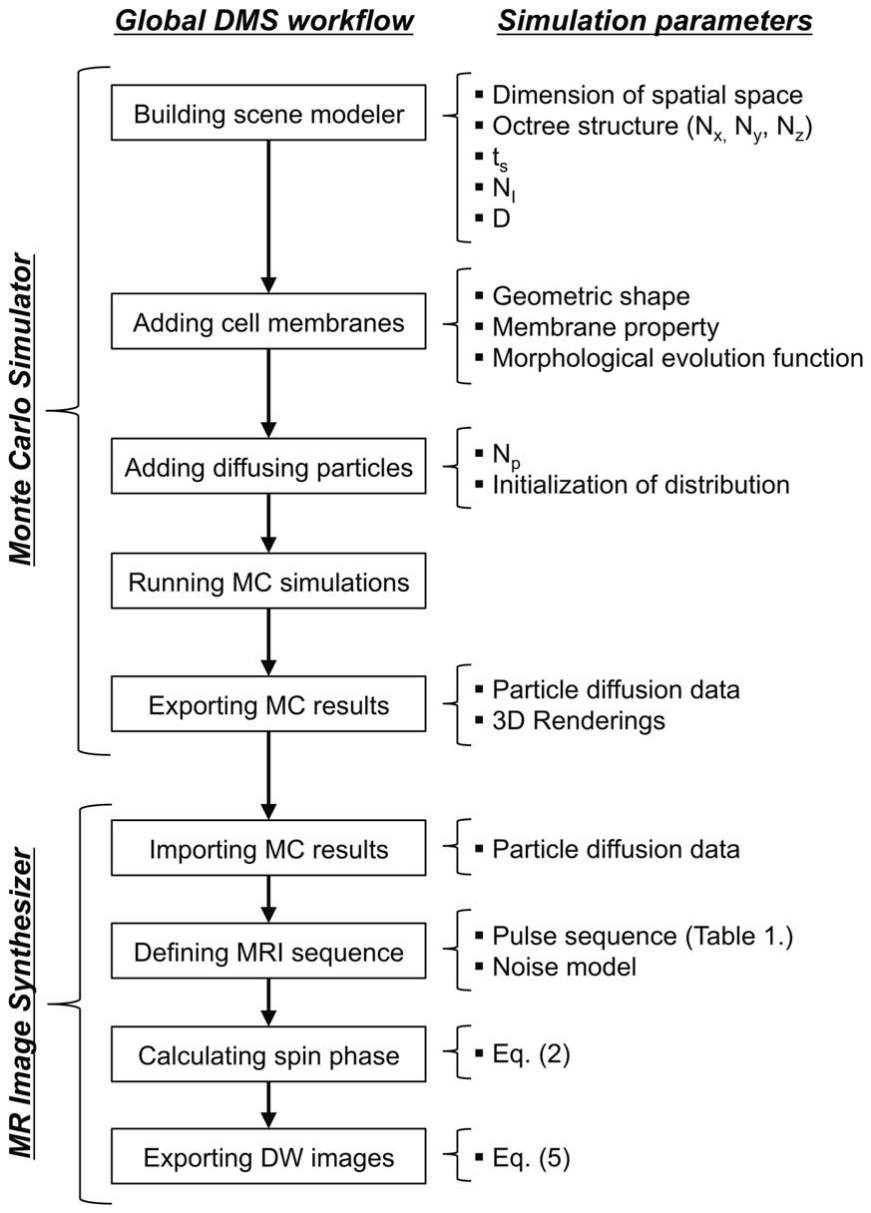
The general overview of DMS simulation procedure and module parameters.

DMS is developed in C++ using an object-oriented design, and it supports multi-threading technique for large-scale simulations on water diffusion in complex environment simultaneously using high spatial and temporal resolution. Fig. 1 illustrates the global workflow of DMS which is composed of two main stages: (i) a random walk Monte Carlo simulator capable of simulating the diffusion of water molecules in an arbitrary simulation environment; and (ii) an MR image synthesizer dedicated to create DW images among various designs of MRI pulse sequences. The concepts for the principle components shown in Fig. 1 are described in the following sections.

### Monte Carlo Simulator

#### Scene modeler

This module contains the essential parameter settings for MC simulations, including the spatial dimensions, the spatial subvolumes (see below), the temporal resolution (i.e. the simulation time step, *t_s_*), the number of iterations (*N_I_*), and the global diffusion model. The scene modeler acts as an interface between the users and the DMS system. It enables users to construct a virtual tissue environment by adding cell membranes and diffusing particles (i.e. water molecules), and to control the start-up of MC simulations. It also contains the functions to save the results of particle diffusion data (e.g. the trajectory and probabilistic density function of water molecules) as well as to animate and visualize the dynamic events of MC simulations.

#### Cell membrane

The cell-membrane module comprises multiple features in order to capture the characteristics of neuronal cells. To mimic various cell types with heterogeneous shapes and sizes, we developed a mesh factory that utilizes triangles as surface elements to form a mesh. The mesh factory can produce mixed networks of geometries such as ellipsoidal, star-shaped, and cylindrical meshes to mimic neural architecture including neurons, glial cells, and axons. The cross sections of axons can be arbitrary shapes and the axonal projections can be any 3D curves. Therefore, DMS has the capability of simulating complex axonal configurations at various states (e.g. bending, beading [51], or degeneration [52]). Moreover, since we incorporated each cell-membrane mesh with a dynamic morphological evolution function, DMS is able to simulate the sequential changes of tissue shapes including expansion, shrinkage, and deformation for modeling different tissue status. The basic properties of cell-membrane layers including the permeability and the type of particle-to-membrane interaction are fully adjustable. Furthermore, the model describing specific diffusion behavior can be designed to the cell-membrane layers. The characteristic of polar membrane layer [50], for instance, has been customized to evaluate the biphasic water diffusion model in this article. For the current DMS, the particle-to-membrane interaction is modeled by total internal reflection rule, which means that the angle of incidence and reflection is identical, and the membrane permeability is modeled using the transmission probability [52], [53], [54].

#### Diffusing particle

We modeled diffusing particles as random walkers. DMS allows users to determine the number (*N_p_*) and initial distributions of particles, which can be (i) regulated by the intra- (*f_i_*) and extracellular (*f_e_* = 1−*f_i_*) fractions, (ii) randomly allocated in the simulation scene (i.e. *f_i_* is proportional to the global cellular volume), or (iii) located at a specific region or location defined by users. The root-mean-squared (RMS) displacement (*r*) of the particle is scaled to the diffusion coefficient (*D*) of the associated tissue compartment and *t_s_* based on the Einstein equation:
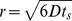
(1)


The direction of diffusion is randomly chosen from a pre-allocated lookup table which included uniform and symmetric orientations obtained using electrostatic repulsion algorithm [55]. The default orientation scheme has a total number of 8,000 directions, where 4,000 orientations are independent. The average/minimum/maximum/standard deviation angles between the neighboring directions are 2.51/1.52/3.97/0.07 degrees, respectively. For each simulation step, a particle updates its spatial position following a series of potential interactions with cell membranes: (i) It may penetrate through the interacting membrane respecting the permeability. (ii) It may move according to the models of particle-to-membrane interactions. (iii) *D* may be altered if an individual membrane model is introduced. For the case of polar membrane layer model, *D* is modified during the transition between the biphasic diffusion pools [50].

#### Spatial subvolume

**Figure 2:**
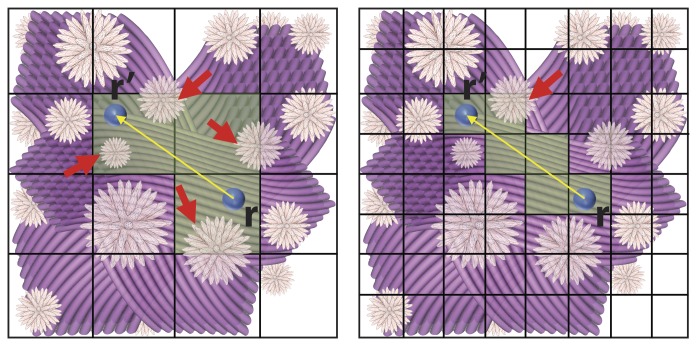
An illustration of the octree structure implemented in DMS. In this simple example, we created groups of fiber bundles and star-shaped cells inside a simulation space which was partitioned using *N_x_*×*N_y_*×*N_z_* of 4×4×1 (left) and 8×8×1 (right). For a water molecule (represented by the blue sphere) diffusing from *r* to *r’*, the need to check for the possible interactions along the path of diffusion can be reduced to the local subvolumes, as shown by the regions colored in green. The number of potential interacting objects to be processed, i.e. the cells indicated by the red arrows, can be decreased systematically.

DMS employs an octree encoding technique by which the global MC simulation space is partitioned into *N_x_*×*N_y_*×*N_z_* subvolumes using a 3D grid. Each subvolume contains a subset of cell-membrane meshes and diffusing particles. Therefore, knowing a particle’s position (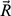), the time required searching and processing any potential interactions can be dramatically decreased via the direct assess to the objective cell membranes simply in the local spatial subvolume rather than the entire simulation space. Fig. 2 illustrates the concept of octree structure of DMS.

### MR Image Synthesizer

#### Spin

The outputs of the Monte Carlo simulator are particle diffusion data, which are imported to the MR image synthesizer and endowed with spins. Each spin stores its phase calculated using the following equation:
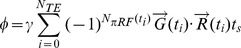
(2)


In Eq. (2), *φ* denotes the spin phase; γ is the gyromagnetic ratio; *N_TE_* is the iteration count at TE (i.e. *N_TE_* = TE/*t_s_*). For a given time point *t_i_*, where *t_i_* = *i*×*t_s_*, *N_πRF_*(*t_i_*) denotes the accumulated counts of refocusing radiofrequency (RF) pulses, 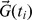 is the gradient vector derived from an NMR pulse sequence (see below), and 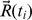 is obtained from particle’s diffusion trajectory. Particle diffusion data also provide additional statistics relating to cell membranes. For the biphasic diffusion model [50], we can segregate the global particles into fast and slow diffusing particles according to their fractions of residence time within the polar membrane layer. Then, the spin phases for two groups of particles can be calculated individually using Eq. (2). This may decouple the dMRI signal into its fast and slow diffusion pools, and thus may help to investigate the impact of the polar membrane layer.

#### NMR sequence

DMS has modeled a variety of MRI pulse sequences by regulating the timings of RF and gradient pulses. Table 1 summarizes the pulse sequences and adjustable parameters available in DMS. Gradient shapes including rectangles, trapezoids, and oscillating waves have been implemented, and can be extended to fit any designs. The imaging gradients, e.g. slice selection, phase encoding, and readout gradients, are optional to be included in an NMR sequence. The echo time is automatically calculated according to the user-specified pulse sequence and related parameters. The diffusion-sensitizing factor, i.e. *b*-value, is determined by the following equations:
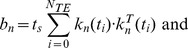
(3)

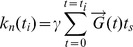
(4)


**Table 1:** The list of available NMR sequences in DMS.

NMR Sequence	Gradient waveform	Parameters
Single PGSE [69]	rectangle; trapezoid	*G*; *S_R_*; δ; Δ; TE
STE [70]	rectangle; trapezoid	*G*; *S_R_*; δ; Δ; TE
Bipolar STE [71]	rectangle; trapezoid	*G*; *S_R_*; δ; Δ; TE
Twice-refocused spinecho [72]	rectangle; trapezoid	*G*; *S_R_*; δ; Δ; TE
Multiple PGSE [73], [74]	rectangle; trapezoid	*G*; *S_R_*; δ; Δ; T_M_; *N_GP_*; TE
Multiple STE [75]	rectangle; trapezoid	*G*; *S_R_*; δ; Δ; T_M_; *N_GP_*; TE
Bipolar double STE [63]	rectangle; trapezoid	*G*; *S_R_*; δ; Δ; T_M_; TE
OGSE [76], [77]	sine; double-sine; cosine	*G*; T (*f*); *N_GO_*; TE

Abbreviations: PGSE, pulsed gradient spin echo; STE, stimulated echo; OGSE, oscillating gradient spin echo; *G*, gradient magnitude; *S_R_*, gradient slew rate; δ, DW gradient duration; Δ, DW gradient separation; TE, echo time; T_M_, mixing time; *N_GP_*, number of DW gradient pairs; T, period; *f* ( = 1/T), frequency of oscillation; *N_GO_*, number of gradient oscillations.

In Eq. (3), *b_n_* represents the *b*-value for the *n*th DW gradient orientation.

#### MR image

This module is created to integrate the spin phases and to synthesize DW images. The noise model, e.g. complex Gaussian noise, can be added to the synthesis data at both real and imaginary channels. The DW signal of an MRI voxel, *S*(*v*), is calculated by numerical integration using the following equation:
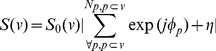
(5)


In Eq. (5), *S*_0_(*v*) is the signal intensity without diffusion-weighting for the voxel *v*; *φ_p_* is the accumulated phase of the *p*th particle calculated using Eq. (2), and *N_p,p⊂v_* is the amount of particles located inside *v* at TE; *η* denotes the complex noise term.

### Benchmark Experiments

We performed four benchmark simulation experiments using DMS as follows:

#### A. Evaluation of computing performance

(a) We ran MC simulations using different sizes of spatial subvolumes to study the impact on the computing time. We placed a network of cells containing 15×15×15 mesh-based spheres with a radius of 5 µm in the simulation space (150×150×150 µm) and varied *N_x_*×*N_y_*×*N_z_* from 10×10×10 to 50×50×50. For each case, MC simulations were repeated ten times using *N_p_* = 10^4^, *N_I_* = 10^3^, *t_s_* = 10 µs, and *D* = 10^−3^ mm^2^/s. Diffusing particles were all initialized inside the confined spheres to produce an adequate amount of particle-to-membrane interactions. (b) We evaluated the computing efficiency of DMS using basic simulation experiments on dMRI of WM fibers. We constructed a hexagonal array of 400 impermeable fibers with diameter, center spacing, and length of 10, 10.1, and 250 µm, respectively. Each fiber was formed with a cylindrical mesh using 40 triangles. The MC simulation parameters were: *N_p_* = 10^4^, *N_I_* = 7,000, *t_s_* = 10 µs, *D* = 2×10^−3^ mm^2^/s, and *N_x_*×*N_y_*×*N_z_* = 50×50×50. Diffusing particles were randomly distributed in the simulation scene. For the dMRI signal synthesis, we applied a PGSE sequence using *G* = 40 mT/m, *S_R_* = 200 T/m/s, δ/Δ/TE = 31.7/37.7/70 ms, *b*-value = 2,600 s/mm^2^, and a uniform DW gradient scheme of 100 orientations. The experiments were repeated for ten times. For both (a) and (b), the computer was equipped with a 2.66 GHz Intel Core 2 Duo processor and a 4 GB 1067 MHz DDR3 memory. For simplicity, the simulations were run using a single thread.

#### B. Multiple scattering diffusion imaging

We used DMS to simulate the conventional dMRI experiments using a single PGSE sequence as well as the multiple scattering diffusion imaging (MSDI) experiments using a double PGSE sequence. The purpose was to demonstrate that DMS could reproduce the simulation results shown in the literature [39]. Accordingly, we created a simulation space (460×400×1,000 µm^3^) where a bundle of parallel impermeable fibers with a diameter and length of 19 and 1,000 µm respectively were placed on a 24×24 hexagonal lattice using a center spacing of 19.1 µm. Each of the fibers was modeled using a cylindrical mesh built by 40 triangles. We performed random walk MC simulations using *N_p_* = 5×10^5^, *N_I_* = 10^5^, *t_s_* = 5 µs, and *N_x_*×*N_y_*×*N_z_* = 160×160×20. *D* ( = 2×10^−3^ mm^2^/s) was assumed to be equal in the intra and extracellular spaces. Synthetic dMRI datasets were then collected using both single and double PGSE sequences with δ/Δ/T_M_ = 2/200/0 ms. A range of *G* (*S_R_* = 5,000 T/m/s) from 0 to 1,200 mT/m with a 10 mT/m increment were applied for the single PGSE, and from 0 to 600 mT/m with a 5 mT/m increment were used for the double PGSE. Note that the simulations reflected the case of a preclinical MRI system. The DW gradients were applied along the direction perpendicular to the fiber axis. In addition, to investigate the influence of *N_p_* on the simulated DW signal, we varied *N_p_* from 10^5^ to 5×10^5^ for the case of single PGSE sequence.

#### C. Biphasic water diffusion model

We used DMS to study the variations of ADC caused by cell swelling and polar membrane layer [50]. We prepared ten simulation spaces with the same dimension of 100×100×100 µm^3^, where each contained a hexagonal network of spherical cells with a specific radius (*R*) increased from 2.40 to 2.58 µm in 0.02 µm increments. Each of the simulation was filled with 9,200 cells using a fixed center spacing of 5.2 µm. Thus, the simulation settings produced ten intracellular volume fractions (i.e. *f_i_*) ranging from 53.27% to 66.18%. For each case, two separate MC simulations were performed (i.e. 20 MC simulations in total): In the first part, we assumed that *D* ( = 1.2×10^−3^ mm^2^/s) was a constant for the entire simulation space. In the second part, we applied the biphasic diffusion model to characterize cell membranes by polar membrane layers. The inner and outer region of the membrane-bound layer represented the slow (*D_slow_* = 0.4×10^−3^ mm^2^/s) and fast (*D_fast_* = 1.2×10^−3^ mm^2^/s) diffusion pool, respectively. Here, we assumed that the cells were impermeable and the thickness of the membrane-bound layer was 40 nm on each side of the membrane. The selection of *D*, *D_slow_*, *D_fast_*, and layer thickness was based on the inferences proposed by Le Bihan [50]. The global MC simulation parameters were *N_p_* = 10^6^, *N_I_* = 15,500, *t_s_* = 5 µs, and *N_x_*×*N_y_*×*N_z_* = 100×100×100. The RMS distances were 0.19 and 0.11 µm for *D_fast_* and *D_slow_*, respectively.

For each of the MC simulations, two noise-free synthetic dMRI datasets were collected using single PGSE sequences: Firstly, the DT datasets were synthesized using a single shell *q*-space sampling scheme of 80 gradient orientations at a *b*-value of 1,000 s/mm^2^, where δ/Δ = 21/27 ms, *G* = 40 mT/m, and *S_R_* = 200 T/m/s. We performed DT reconstruction to estimate the ADC [4]. Secondly, the DW signal along *x*-, *y*-, and *z*-axis were synthesized at 51 *b*-values linearly increased from 0 to 5,000 s/mm^2^, which were achieved by fixing δ/Δ = 2/70.5 ms and varying *G* (*S_R_* = 5,000 T/m/s). The normalized diffusion signal attenuation along each axis was then fitted using a biexponential function given as follows:
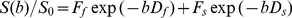
(6)


In Eq. (6), *F* and *D* were the volume fractions and diffusion coefficients associated with the fast and slow diffusion phases. The mean and standard deviation were calculated for the model parameters derived from the three axes.

#### D. HARDI and fiber-tracking applications

We applied DMS to simulate high angular resolution diffusion imaging (HARDI) of different WM fiber configurations for evaluation of fiber-tracking algorithms. Here, we created crossing, kissing, and branching fibers in the separated simulation spaces with sizes of 110×190×150, 110×190×150, and 200×210×185 µm^3^, respectively. Each fiber had a diameter of 5 µm and no permeability. The parameters for the MC simulations were: *N_p_* = 10^6^, *N_I_* = 8,000, *t_s_* = 10 µs, *D* = 2×10^−3^ mm^2^/s, and *N_x_*×*N_y_*×*N_z_* = 150×150×50. We synthesized DW images using a conventional PGSE sequence with *G* = 40 mT/m, *S_R_* = 200 T/m/s, δ/Δ/TE = 34.75/40.75/80 ms, and *b*-value = 4,000 s/mm^2^. The uniform HARDI sampling scheme consisted of 200 unique orientations [56]. These parameters were chosen to conform to the clinical MRI system. Simulated DW images were generated using a grid volume that produced a single slice with an in-plane resolution of 5×5 µm^2^. For each fiber configuration, we reconstructed the fiber orientation distribution function (fODF) using the sharpening deconvolution transform (SDT) with a spherical harmonic order of 6 and a regularization factor of 0.006 [46]. DT analyses were also performed to obtain the fractional anisotropy (FA) maps, which were used to create mask images for fiber tracking. Both deterministic and probabilistic fiber tractography were obtained via the streamline fiber-tracking algorithm, with a forward step increment of 1.25 µm (i.e. one-fourth of the in-plane resolution), an aperture angle of 30°, and 10 seeds per voxel [33], [34], [57].

## Results

### Modeling Neural Microstructures

**Figure 3:**
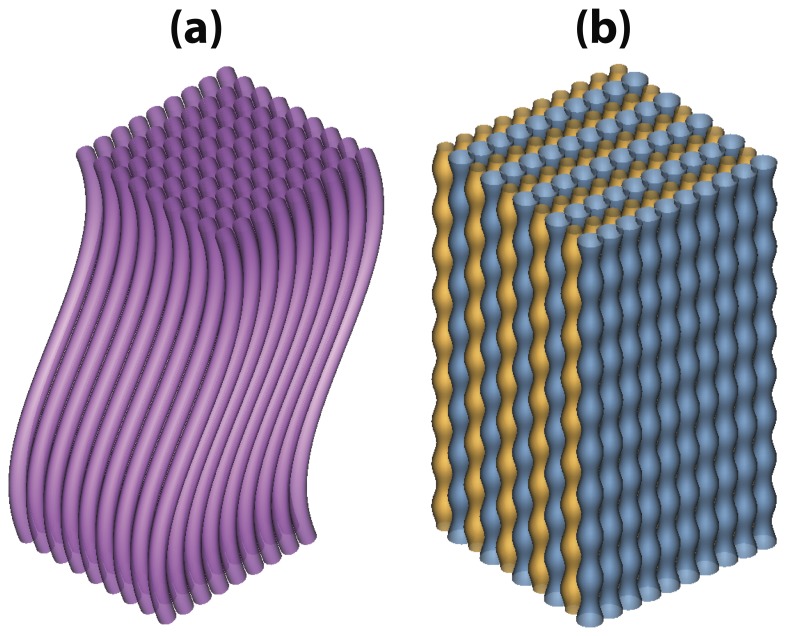
The 3D renderings of the bending (left) and beading (right) axon models.

**Figure 4:**
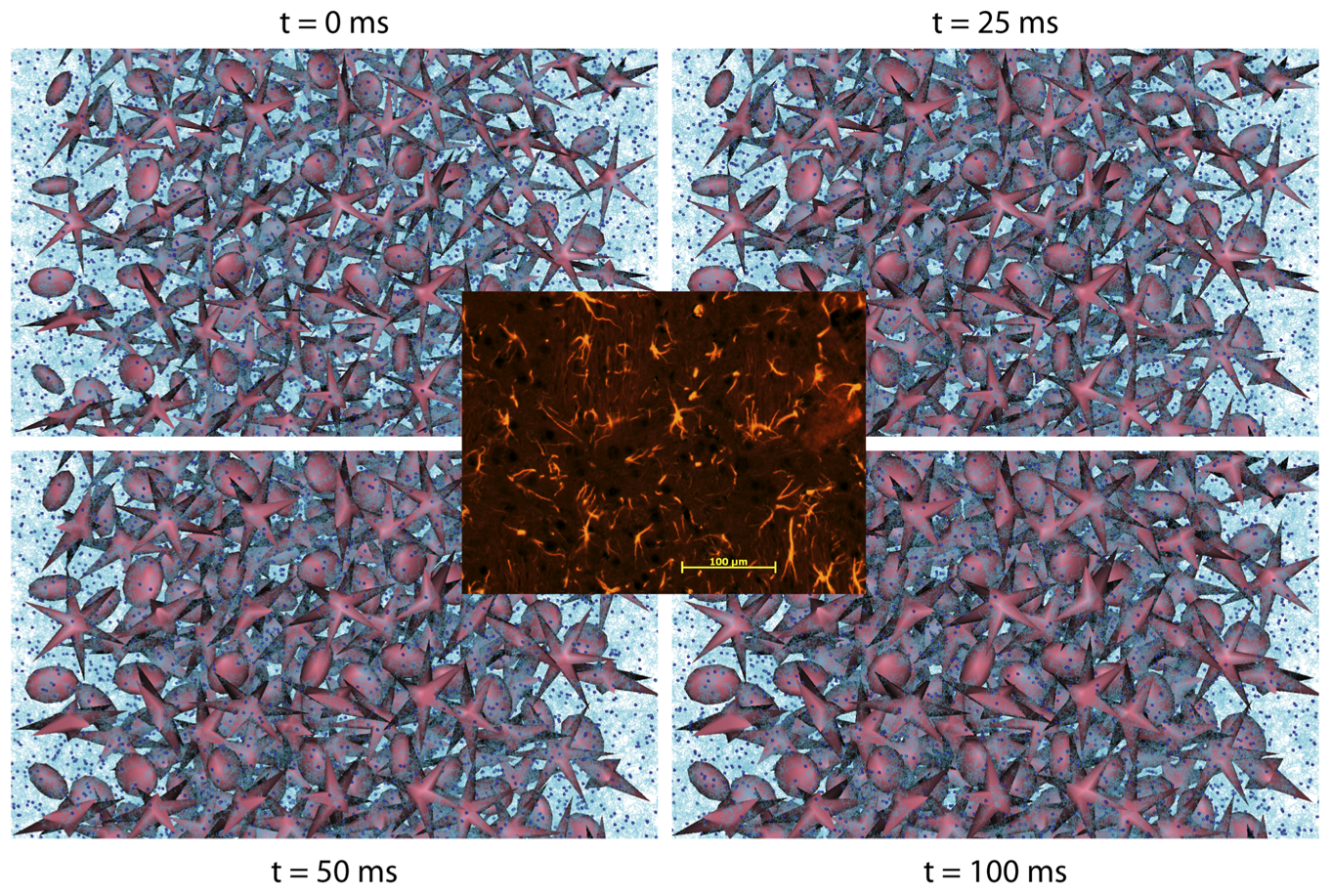
Modeling the neural medium using DMS. The image at the center shows the immunostaining of neural tissues, and the others are the 3D renderings of simulated glial cells (colored in red) at different time points. The cell gradually expanded due to the effect of dynamic morphological evolution function. Dark blue spheres and light blue curves represented the diffusing particles and their motion trajectories.

Figs. 3–4 are two examples of cell membrane models showing the potential of DMS to simulate different architecture and conditions of neural tissues. Fig. 3(a) shows a bundle of bending axons, and Fig. 3(b) illustrates the compact beading axons modeled using two networks of axons. Fig. 4 shows a virtual neural substrate simulated via DMS. The image in the center of Fig. 4 was a fluorescence micrograph obtained from immunostaining of a healthy mouse brain, where the glial cells were dyed using anti-GFAP [58]. We used DMS to mimic the micrograph of the neural tissue in 3D. Moreover, since we incorporated each cell with a dynamic morphological evolution function to simulate cell swelling, the cells were expanding from the smallest sizes at the beginning (t = 0 ms) to the largest sizes at the end (t = 100 ms) of the simulation. The movie animation of the dynamic cell swelling can be found in the online version (Video S1).

Table 2 summarizes the results of elapsed times for the MC simulations using different numbers of spatial volumes. The results revealed that using a large octree structure (*N_x_*×*N_y_*×*N_z_*) could significantly improve the computing efficiency. For a complete DMS simulation on dMRI of WM fibers, the average times required by MC simulations and MR image syntheses were 396.7±5.5 and 108.8±2.2 seconds, respectively. Note that the results for the two experiments above were obtained without the application of the multithreading feature that is supported by DMS.

**Table 2:** The elapsed times (mean ± standard deviation) for the MC simulations using different dimensions of spatial subvolumes.

*N_x_×N_y_×N_z_*	10×10×10	20×20×20	30×30×30	40×40×40	50×50×50
**Elapsed time (s)**	973.15±0.19	252.91±0.12	121.21±0.08	114.01±0.04	110.61±0.07

### Multiple Scattering Diffusion Imaging

**Figure 5:**
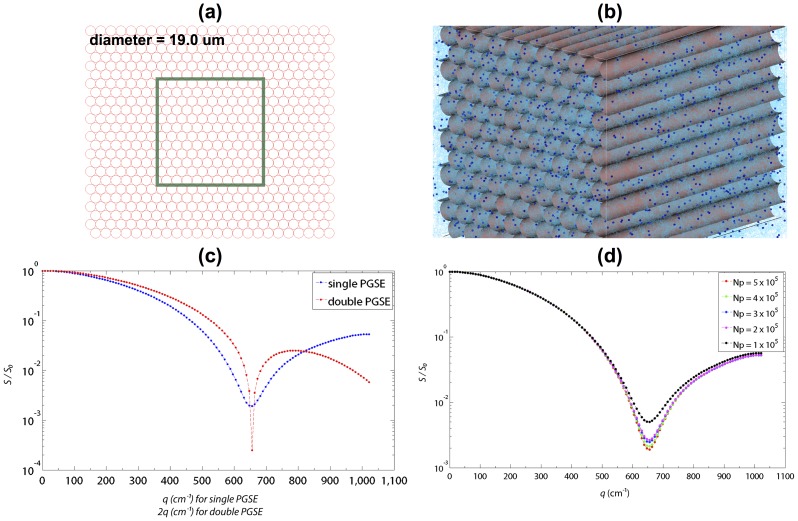
The DMS simulation of MSDI. (a) The transverse view of the hexagonal network of mesh-based cylindrical fibers, which had a diameter of 19 µm. (b) A snapshot of the MC simulation scene illustrating the zoomed area within the green square in (a), where the dark blue spheres and light blue curves are the diffusing particles and their corresponding diffusion trajectories, respectively. (c) Plots of the diffusion diffraction patterns obtained from single and double PGSE pulse sequences. (d) Plots of dMRI signal attenuation under different *N_p_*.

Fig. 5(a) shows the transverse view of the hexagonal network of mesh-based cylindrical fibers, and Fig. 5(b) shows a snapshot of the MC simulation scene. Fig. 5(c) shows the results of diffusion signal attenuation obtained from the single and double PGSE measurements. For comparison, the signal decay was plotted against 2*q* for the double PGSE. The first diffusion diffraction trough was observed at the *q*-value of 655.7 cm^−1^ for both sequences, corresponding to an estimated fiber diameter of 18.6 ( = 1.22×10^4/^655.7) µm based on Callaghan theory [59]. The results were closed to the actual diameter of 19 µm defined in the MC simulations. Fig. 5(d) shows the dependency of dMRI signal synthesis on *N_p_*. The diffraction trough became less obvious while *N_p_* decreased.

### Biphasic Water Diffusion Model

**Figure 6:**
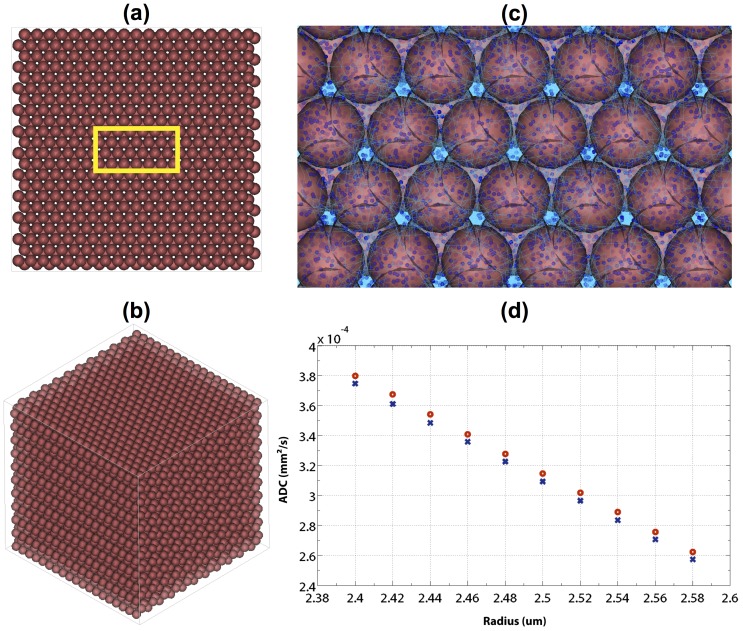
The DMS simulation of water diffusion in virtual neuronal cells. (a) A transverse section of the simulation scene which contains a hexagonal network of cells modeled by spheres (*R* = 2.58 µm). (b) A global view of the simulation space illustrating the arrangement of the cells. (c) A zoomed region of (a) showing the 3D rendering of cells (colored in red), diffusing particles (the spheres in deep blue) and their motion trajectories (the curves in light blue). (d) The ADCs estimated using the DT model for the case of constant diffusivity (red circle) and biphasic water diffusion model (blue cross).

Figs. 6(a–c) illustrate the 3D renderings of the MC simulations considered in this section, and Fig. 6(d) is the plot of ADCs against cell sizes. As expected, we found that the ADC decreased when the cell size increased, and additional reduction in ADC was produced owing to the effect of membrane-bound layer. Tables 3–4 summarize the model parameters derived from the biexponential curve fitting to the synthetic dMRI signal. From both tables, we observed that when the cell size increased, *F_f_* decreased continuously, and an opposite trend was found for *F_s_*; meanwhile, increasing cell size appeared to result in a decrease in *D_f_* and an increase in *D_s_*.

**Table 3:** Biexponential fitting parameters (mean ± standard deviation) for the case of constant diffusivity (*D* = 1.2×10^−3^ mm^2^/s).

*R*	*F_f_*	*D_f_*	*F_s_*	*D_s_*
**2.40**	0.5196±0.0017	0.9333±0.0194	0.4810±0.0017	0.0083±0.0005
**2.42**	0.5054±0.0009	0.9304±0.0237	0.4952±0.0009	0.0089±0.0003
**2.44**	0.4929±0.0021	0.9195±0.0240	0.5076±0.0021	0.0090±0.0009
**2.46**	0.4805±0.0012	0.9097±0.0294	0.5201±0.0013	0.0092±0.0004
**2.48**	0.4653±0.0004	0.9053±0.0299	0.5353±0.0003	0.0100±0.0001
**2.50**	0.4537±0.0016	0.8933±0.0306	0.5468±0.0016	0.0096±0.0003
**2.52**	0.4383±0.0014	0.8901±0.0322	0.5623±0.0014	0.0102±0.0004
**2.54**	0.4243±0.0024	0.8809±0.0337	0.5763±0.0024	0.0105±0.0005
**2.56**	0.4094±0.0011	0.8697±0.0383	0.5913±0.0011	0.0109±0.0003
**2.58**	0.3960±0.0014	0.8594±0.0442	0.6047±0.0014	0.0109±0.0003

Unit: *R*, µm; diffusivity, 10^−3^ mm^2^/s.

**Table 4:** Biexponential fitting parameters (mean ± standard deviation) for the case of biphasic water diffusion model (*D_fast_* = 1.2×10^−3^ & *D_slow_* = 0.4×10^−3^ mm^2^/s).

*R*	*F_f_*	*D_f_*	*F_s_*	*D_s_*
**2.40**	0.5183±0.0008	0.9151±0.0209	0.4823±0.0008	0.0089±0.0003
**2.42**	0.5063±0.0010	0.9056±0.0227	0.4943±0.0009	0.0091±0.0004
**2.44**	0.4919±0.0022	0.9005±0.0242	0.5087±0.0021	0.0096±0.0008
**2.46**	0.4812±0.0011	0.8899±0.0285	0.5194±0.0011	0.0093±0.0003
**2.48**	0.4660±0.0013	0.8835±0.0261	0.5346±0.0013	0.0101±0.0003
**2.50**	0.4526±0.0005	0.8743±0.0314	0.5481±0.0006	0.0100±0.0002
**2.52**	0.4388±0.0024	0.8676±0.0313	0.5618±0.0023	0.0103±0.0005
**2.54**	0.4246±0.0022	0.8558±0.0321	0.5761±0.0022	0.0107±0.0005
**2.56**	0.4095±0.0009	0.8465±0.0391	0.5912±0.0009	0.0110±0.0004
**2.58**	0.3962±0.0019	0.8333±0.0427	0.6045±0.0018	0.0110±0.0005

Unit: *R*, µm; diffusivity, 10^−3^ mm^2^/s.

### HARDI and Fiber Tracking Applications

**Figure 7:**
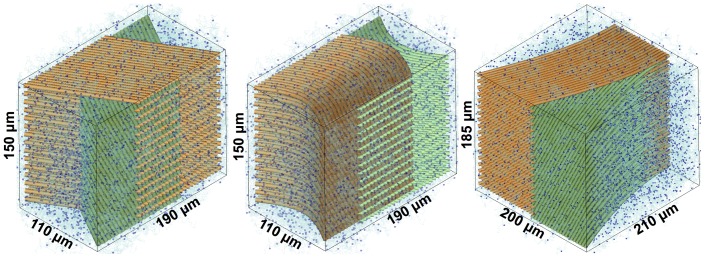
We used DMS to combine two networks of fibers (colored in green and orange) for mimicking crossing (left), kissing (middle), and branching (right) WM fibers of human brains. Dark blue spheres and light blue curves illustrated a subset of diffusing particles and their motion trajectories.

**Figure 8:**
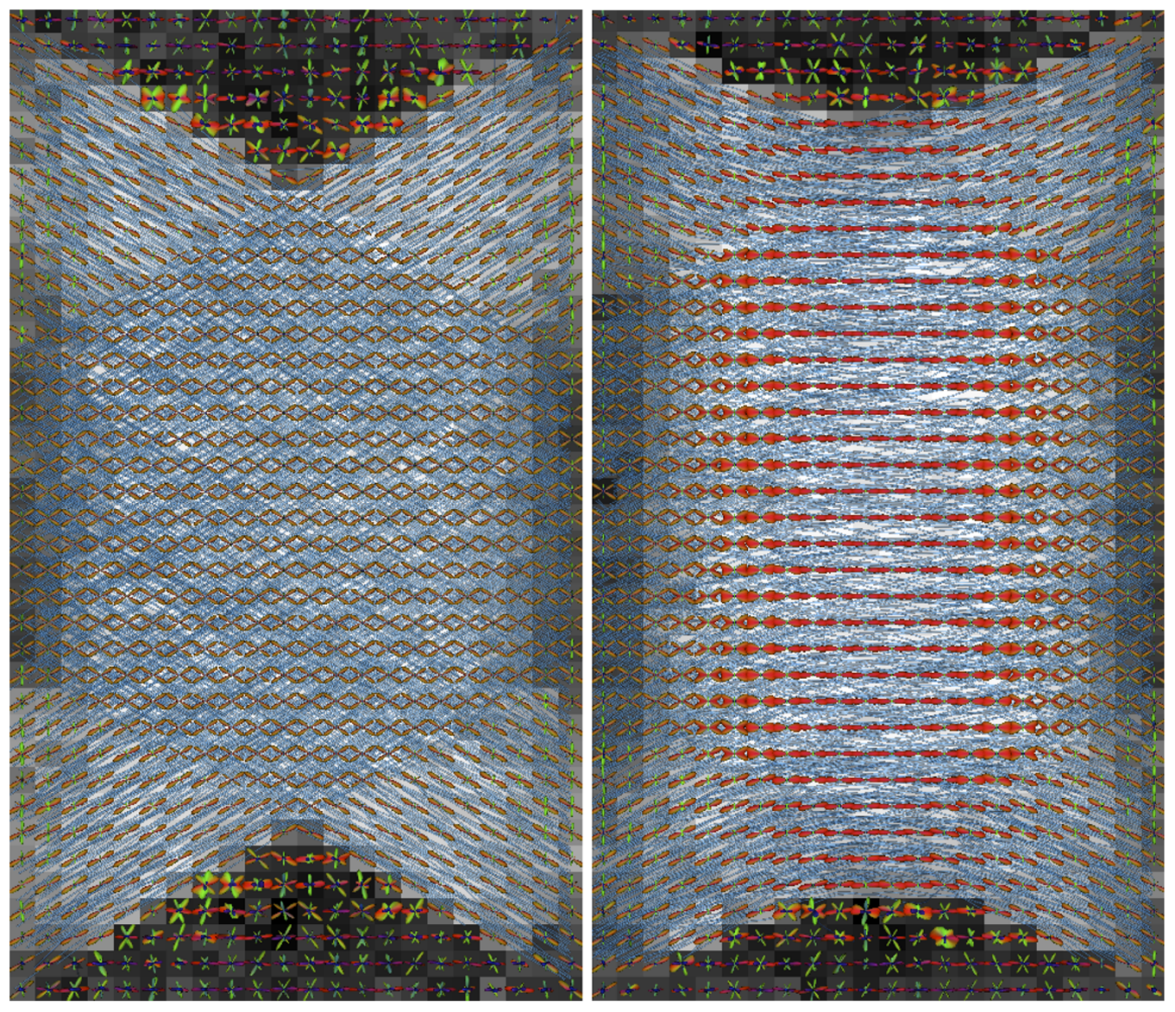
Fiber tractography of the simulated crossing (left) and kissing (right) fibers. The fiber tracts were represented by cylinders colored in blue, and the SDT-fODFs were color-coded depending on orientations (red: left-right, green: top-down, blue: inferior-superior).

**Figure 9:**
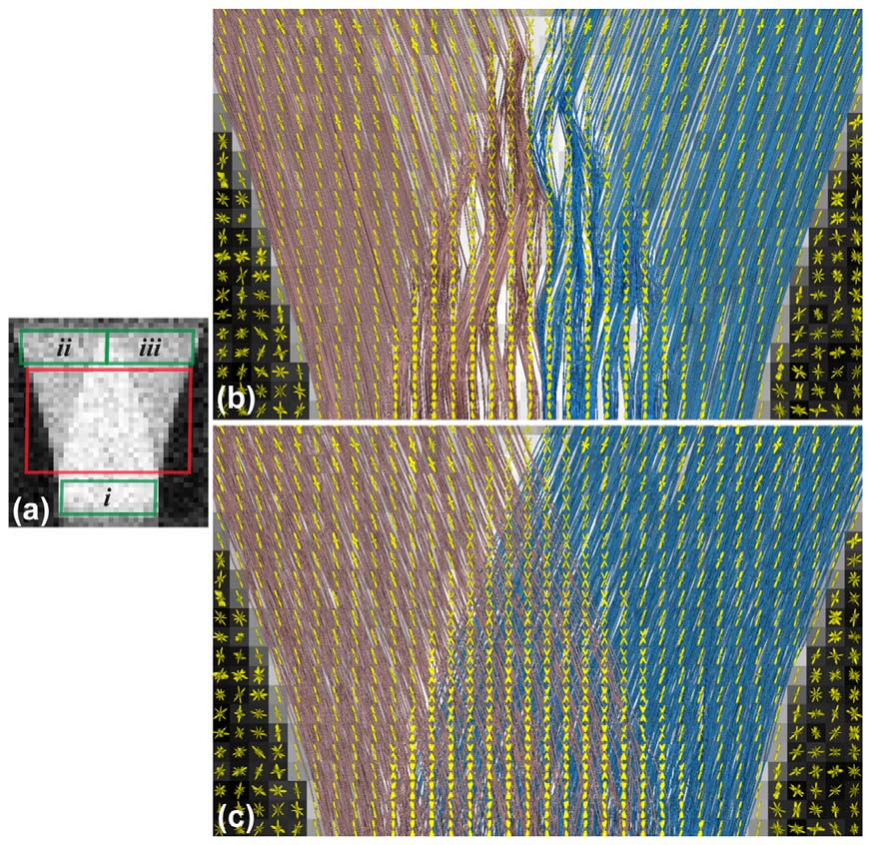
Fiber tractography of the simulated branching fibers. (a) The DT-FA map and the regions of interests defined for clustering the fiber tracts. (b) Fiber tracking using the deterministic method. (c) Fiber tracking using the probabilistic method. The SDT-fODFs were colored in yellow.

Fig. 7 shows the geometric designs of crossing, kissing, and branching fibers. For each case, two networks of fibers were arranged in an interleaved fashion. Figs. 8–9 show the results of fODFs and streamline fiber tractography superimposed onto the FA images. In Fig. 8, we found that SDT generated different fODF patterns in the regions of crossing and kissing fibers, and therefore the ground-truth fiber pathways were successfully differentiated using the probabilistic fiber-tracking algorithm. Fig. 9 shows the comparison between the deterministic and probabilistic methods for the case of branching fibers. As shown in Fig. 9(a), the fiber tracts passed through the areas “*i*” & “*ii*” were colored in red, and through “*i*” & “*iii*” were colored in blue. Figs. 9(b–c) illustrated that the deterministic approach resulted in ambiguous fiber pathways at the region where the ground-truth fiber tracts tended into two distinct directions, while the probabilistic approach presented a good agreement with the ground-truth fiber configuration.

## Discussion

In this article, we present the global architecture and the potential applications of DMS. The spirit of DMS is to provide a platform for users to perform various basic dMRI simulations as well as for advanced developers to customize plug-in functions and adapt the DMS modules to the study of interests. While being conceptually similar to previous MC-based simulators [53], [54], [60], the overall design of DMS adds values to other systems in terms of performance, usability, integrity, and extensibility and should therefore prove a useful addition to the field. According to our results, we demonstrated that DMS was already applicable to address a variety of topics (e.g. tissue modeling, diffusion biophysics, pulse sequence optimization, diffusion reconstruction model, fiber-tracking and post-processing techniques) and ready to go beyond experiments to provide new scientific insights. The first generation of DMS will be released through a dedicated BrainVISA toolbox (http://brainvisa.info).

### Modeling Neural Microstructures

The most difficult task of dMRI simulations is likely to be the construction of a 3D simulation scene that resembles a biological environment, as neural tissues contain various types of cells with diverse sizes and shapes, such as glial cells, neuron bodies, axons, dendrites. Even for the WM fibers, tissue components such as cell nucleus, microfilament, microtubule, or myelin sheath are usually ignored in dMRI simulations. It is not practical to model each cell type mathematically since analytical expressions for complicated geometries may not exist. Furthermore, in reality the spaces between cells are extremely narrow (∼tens nm), it is a great challenge to represent tightly packed configurations all together in 3D. In our benchmark experiments, we have shown that DMS is able to create various axon fiber configurations and complex networks of neuronal cells extracted from the micrograph of brain tissues; meanwhile, it is also possible to simulate the dynamic events of tissues such as cell swelling. However, it is still problematic to construct randomly distributed but highly compact cells. To address this issue, we are currently in the process of programming an automatic algorithm to generate meshes without overlapping so as to mimic complex neural media (e.g. brain gray matter).

### Computing Performance and System Stability

We have shown that increasing the octree resolution can substantially decrease the computation time in our DMS experiments. However, it is important to note that keeping increasing the number of partitions may not necessarily gain in computing efficiency. Actually, optimizing the size of octree structure could be a difficult task since it depends on multiple factors including the resolution of cell-membrane mesh, the RMS displacement of diffusing particle, and the available computer memory. Nevertheless, the current method can be ameliorated using an adaptive algorithm that takes the density of mesh element into account. It means that the regions containing more mesh elements will be divided into finer subvolumes. Likewise, for those containing few or no polygons will be merged with their neighbors. We expect that it will be helpful to improve the efficiency and save the memory consumption.

Hall and Alexander have proposed that the simulation complexity ( = *N_p_*×*N_I_*) for a simple substrate of cylinders has to be greater than 10^8^ to avoid statistical errors [54]. It is obvious that the system complexity determines the duration of the simulation. Our results support that DMS can complete similar simulations within 10 minutes for a system complexity of *N_p_* ×*N_I_* ∼10^8^. In the simulation of cell-membrane properties, for instance, since the layer thickness of the polar membrane interface is on a nanometric scale [50], the time step (i.e. *t_s_*) needs to be short enough to reach an adequate temporal resolution and prevent from the jumping effect of RMS distance [61]. Consequently, it may cause a great increase in *N_I_* for simulating an actual dMRI experiment that generally spans tens to hundreds of milliseconds. Hence, it reflects the importance of the facility in supporting high computing system for large-scale simulations (i.e. fine tissue architecture, high temporal resolution, large amount of diffusing particles, and complex particle-to-membrane interactions).

In principle, the pre-experiments need to be performed for each dMRI simulation scheme in order to ascertain the appropriate values for the combination of *N_p_* and *N_I_* for reproducibility. It is also clear that the system complexity should vary with the complexity of the substrate and the pulse sequence. While this procedure was beyond the purpose of this study, we directly used the empirical values in our benchmark experiments by choosing adequate combinations of *N_p_* (≥10^6^) and *N_I_* (≥10^3^) to produce a minimum order of system complexity greater than 10^9^.

### Application on Microscopic Diffusion MRI

MSDI is a generalized PGSE technique that has the capacity to explore compartment anisotropy, pore size and shape at microscopic level via its sensitivity to small compartmental dimension [62], [63], [64]. Prior to our MSDI experiments, the accuracy of DMS had been verified via a simple comparison against an analytical model, and the results matched within numerical error (data not shown). Overall, our results supported the validity of DMS and were consistent with the theory proposed by Callaghan [59] and with the data shown by Shemesh et al. [39]. As suggested by the results shown in Fig. 5(d), an adequate number of particles are required for high *q*- or *b*-value dMRI experiments, which is also matched with the conclusion in [53], [54]. Note that in our MSDI simulations, the compact hexagonal arrangement of the impermeable axons approximately formed a restricted extra-axonal space in the transverse plane (see Fig. 5(a)). Hence, the *q*-space estimate of the axon diameter was potentially a mixture value of intra- and extra-axonal sizes. It may be the reason that the real axon size was slightly underestimated in this study.

### Application on Tissue Models

For the experiments on the cell swelling and biphasic diffusion model, overall we observed and verified that cell swelling led to a drop of ADC, which matched with the previous findings on acute ischemic stroke [65] and neuronal activation [10], [50], [66]. Based on the results obtained from the biexponential analyses, we found that *D_f_* and *D_s_* did not alter significantly following cell swelling. The results implied that the variation of volume fractions *F_f_* and *F_s_* mainly drove the variations of diffusion signal attenuation. To the best of our knowledge, this is the first time that the hypothesis of cell-membrane-bound layer is evaluated using large-scale MC simulations with high complexity and exquisite mesh-based geometries. The system complexity has a major impact on the simulation time, which again reflects the importance of computing efficiency. Note that although we could already simulate the effect of polar membrane layer, the RMS distances that we used (0.19 and 0.11 µm) were larger than the layer thickness of 0.04 µm assumed in the literature [50]. This had been proved to affect the results, especially when the diffusing particles were closed to the cell membranes [61], and thus it will require a smaller *t_s_* to clarify this issue. Works are undergoing to introduce more factors such as the membrane permeability to perform more complex and realistic simulations.

### Application on HARDI and Fiber Tractography

In this section, we demonstrated that DMS could produce ground-truth dMRI datasets of different designs of fiber distribution. Numerous conditions can be simulated for evaluation and comparison of diffusion reconstruction models and fiber-tracking algorithms (e.g. modifying the signal-to-noise ratio or the fiber density). Our results shown in Figs. 8–9 revealed that the fiber tractography based on SDT and probabilistic fiber-tracking method could distinguish complex fiber pathways. However, using the deterministic approach produced the false-positive streamlines that did not match with the ground-truth fiber configuration, as illustrated in Fig. 9(b). In addition, a part of fibers passing through the area “*i*” (see Fig. 9(a)) were missing, which implied that the results of the subsequent tract-based processing such as fiber clustering or network analysis could be affected while using the deterministic fiber-tracking algorithm. In fact, the wiggly streamlines also appeared in the probabilistic tractography; nevertheless, compared with the global results, the proportion of the false streamlines was relatively low. Note that although we performed fiber tracking on anisotropic voxels in our experiments, there should not be any severe side effects on the results of fiber tractography. This was because the fibers were all aligned on the plane of isotropic resolution, the anisotropic scale which is in the direction perpendicular to the plane should not strongly influence the fiber-tracking propagation. In summary, our results suggest that the selection of fiber-tracking algorithm is crucial as it may significantly alter the fiber tractography. Also, DMS is suitable to evaluate the intrinsic limitations as well as to optimize the parameters for the diffusion reconstruction models and the fiber-tracking algorithms.

### Future Extension

Further extension of DMS is straightforward due to its framework design. The directions for future developments are as follows: (i) Similar to the idea of Panagiotaki et al. [67], we are going to develop a new 3D rendering technique that aims to build meshes from a series of histological images of biological samples. (ii) DMS currently supports the multithreading technique and the new generation will be compatible with the graphic processing unit (i.e. GPU) for acceleration, as demonstrated by Waudby and Christodoulou [68]. (iii) DMS will be extended to characterize the spin system using Bloch-Torrey equation, and the tissue properties including the spin-lattice (T_1_) and spin-spin (T_2_) relaxation times will be considered for the MRI signal synthesis. This would be helpful in many aspects such as developing dMRI pulse sequences (e.g. designing RF or gradient waveform) and modeling MRI artifacts (e.g. eddy current, cross-term interaction, or magnetic field inhomogeneity).

## Conclusion

DMS is a novel dMRI simulation platform that has a general and flexible framework, which can be used to assess the abilities and limitations of dMRI to image various tissue characteristics over a large range of experimental conditions. We expect that DMS may serve as an essential tool for the development, validation, and optimization of dMRI schemes for different applications as well as for giving insights into the interrelationship between the fundamental diffusion process in biological tissues and the features observed in dMRI.

## Supporting Information

Video S1 **The video shows the MC simulation of water diffusion in a virtual neural substrate where the cells (colored in red) are gradually expanded in order to animate dynamic cell swelling.** The diffusing particles and their motion trajectories are depicted using deep blue spheres and light blue curves, respectively.
(MOV)
